# Permeabilization of *Cryptosporidium* spp. Oocysts in Water, Apple and Carrot Juice by Pulsed Electric Field Technology

**DOI:** 10.3390/foods14122112

**Published:** 2025-06-16

**Authors:** Alejandro Berzosa, Laura Garza-Moreno, Joaquín Quílez, Javier Raso, Ignacio Álvarez-Lanzarote, Juan Manuel Martínez

**Affiliations:** 1 Departamento de Producción Animal y Ciencia de Los Alimentos, Tecnología de Los Alimentos, Facultad de Veterinaria, Instituto Agroalimentario de Aragón (IA2), Universidad de Zaragoza, 50009 Zaragoza, Spain; aberzosa@unizar.es (A.B.); jraso@unizar.es (J.R.); 2 Departamento de Patología Animal, Facultad de Veterinaria, Instituto Agroalimentario de Aragón (IA2), Universidad de Zaragoza, 50009 Zaragoza, Spain; lgarza@unizar.es (L.G.-M.); jquilez@unizar.es (J.Q.)

**Keywords:** *Cryptosporidium*, PEF, inactivation, electroporation, beverage, fruit, vegetable

## Abstract

*Cryptosporidium* spp. oocysts are highly resistant to conventional disinfection methods and have been associated with foodborne outbreaks linked to unpasteurized fruit and vegetable juices. This study aimed to evaluate the effectiveness of Pulsed Electric Fields (PEF) in permeabilizing *Cryptosporidium* oocysts in water, apple juice, and carrot juice. Oocysts were exposed to monopolar square-wave pulses (3 µs) at electric field strengths ranging from 15 to 35 kV/cm, with treatment times up to 180 µs, and application temperatures between 25 °C and 60 °C. Membrane permeabilization was assessed using propidium iodide uptake via fluorescence microscopy and flow cytometry. Results showed that oocyst permeabilization increased with electric field strength, treatment time, and temperature, with up to 90% permeabilization achieved at 35 kV/cm and 45 °C. Carrot juice treatments yielded higher permeabilization levels than apple juice, attributed to greater electrical conductivity and energy input. Temperatures below 60 °C alone had negligible effects, but synergistically enhanced PEF efficacy. These findings demonstrate that PEF, particularly when combined with mild heat, is a promising non-thermal technology for reducing *Cryptosporidium* viability in beverages, offering an effective alternative for improving the microbiological safety of minimally processed juices while preserving sensory and nutritional quality.

## 1. Introduction

*Cryptosporidium* spp. is an intracellular protozoan parasite, of which 42 different species have been recognized. Among these, *C. hominis* and *C. parvum* are most notable, responsible for the majority of human infections [1]. *C. hominis* is primarily an anthroponotic species, although its presence has been sporadically reported in various animals. In contrast, *C. parvum* is a potentially zoonotic species, capable of infecting a wide variety of mammals, including ruminants and humans. It is worth noting that *Cryptosporidium* is a ubiquitous parasite with a low infectious dose (132 oocysts for *C. parvum* and 10–83 for *C. hominis*) and high resistance to disinfectants used for drinking water treatment, making it the most prevalent waterborne protozoan in developed countries [2].

*Cryptosporidium* spp. infects the epithelial cells of the gastrointestinal tract of vertebrates. Its biological cycle is monoxenous, meaning it develops entirely within the same host. Infection occurs orally through the ingestion of food or water contaminated with oocysts, and upon reaching the stomach, four sporozoites are released that invade the enterocytes [3]. Within the intestinal epithelial cells, the parasite undergoes two cycles of asexual reproduction that severely damage the epithelium, followed by a sexual phase that culminates in the formation of oocysts with sporozoites through intracellular sporogony. Most oocysts have a thick wall and are excreted in feces, potentially being ingested by another host, and the cycle begins anew.

The global prevalence of cryptosporidiosis in humans is 7.6%, increasing to 31.5% in developing countries (with an average of 10.4%) and ranging from 0.1% to 14.1% in developed countries, with an estimated average of 4.3% [4]. Cryptosporidiosis is a disease under surveillance in the European Union, although mandatory reporting varies between countries, being obligatory in some member states such as Germany, Spain, Ireland, and Sweden [5]. This disease is considered a global health emergency, especially for children and immunocompromised patients. According to the European Centre for Disease Prevention and Control (ECDC), there was an increase in cases in 2021 compared to previous years, with a total of 4476 cases reported by 24 EU/EEA countries. The most affected age group was children aged 0–4 years, with a notification rate of 6.4 per 100,000.

Although oocysts do not multiply in water, their small size allows them to evade conventional filtration treatments and they can survive for long periods when exposed to chlorine and other chemicals commonly used for drinking water purification [6,7]. Chlorine requires high doses and long exposure times to be effective, yet there is a threshold beyond which it has no additional impact [8]. Ozone, while effective, contaminates the air and is irritating to humans [9]. Distillation can remove up to 99.5% of microbes but is extremely energy-intensive, as it requires boiling water and condensing the vapor making it impractical for large-scale treatment [10]. In the absence of effective treatment, this resilient parasite can survive in drinking water and occasionally causes outbreaks that impact public health [11].

In addition to waterborne transmission, *Cryptosporidium* has gained prominence as a food safety concern, with an increasing number of foodborne outbreaks attributed to its presence. Transmitted via the fecal-oral route, its environmentally resilient oocysts can contaminate a wide range of food products, including fresh produce, unpasteurized juices, and raw milk. Notably, the infectious dose is extremely low, meaning that even minimal contamination poses a substantial health risk [12,13]. The first large-scale foodborne outbreak was reported in the United States in 1996 involving freshly prepared apple juice [13]. A 2005 outbreak in Denmark was traced to a contaminated salad bar, with ingredients like carrots and red peppers identified as the source [14]. More recently, an outbreak in Norway linked to self-pressed apple juice contaminated with *C. parvum* emphasized the microbiological risks associated with artisanal juice production [15]. Collectively, these outbreaks—along with additional cases reported in non-scientific sources—highlight that unpasteurized fruits and vegetables can consistently act as vehicles for *Cryptosporidium* transmission. This underscores the urgent need for effective and alternative safety measures in the production of minimally processed beverages. As the consumption of products such as juices, smoothies, purées, compotes, and fresh-cut fruits and vegetables continues to rise—largely due to their perceived nutritional benefits—the combination of *Cryptosporidium*’s low infectious dose and high environmental resilience presents a persistent and significant challenge to food safety within this category [16,17].

Traditional thermal treatments, such as flash pasteurization, have been shown to achieve significant reductions in oocyst viability. Specifically, heat treatments at 71.7 °C for 10–20 s have been shown to inactivate up to 99.999% [18]. However, reliance on heat treatment can negatively impact the sensory and nutritional qualities of fruit juices, altering flavor, aroma, and vitamin content, which is particularly undesirable in the production of fresh or premium juices marketed for their natural attributes [19]. Non-thermal methods are also under investigation for their potential to preserve nutritional and sensory qualities of juices while ensuring microbial safety. Ultraviolet (UV) irradiation has demonstrated potential to inactivate *C. parvum* in apple cider without altering taste or appearance, but its effectiveness can be significantly reduced in turbid or colored juices where suspended particles shield oocysts from UV exposure, leading to incomplete disinfection [20]. Similarly, chemical treatments using organic acids like malic and citric acid, or oxidizing agents such as hydrogen peroxide and ozone, have shown variable efficacy depending on concentration and exposure time, but they may also affect the organoleptic properties of the juice or leave residual compounds that could alter flavor, pH, or consumer acceptability [21,22]. High hydrostatic pressure (HHP), ultrasounds, and pulsed light technologies are emerging as promising alternatives, achieving significant oocyst reduction with minimal impact on juice quality, but these methods can involve high operational costs, specialized equipment, and may still require optimization for consistent efficacy across different juice matrices and contamination levels [23,24]. Continued research into these inactivation strategies is essential for developing safe, non-thermal juice processing methods that can reliably target *Cryptosporidium* and other protozoan pathogens.

The physical alternative proposed in this study to inactivate *Cryptosporidium* oocysts is Pulsed Electric Fields (PEF). PEF treatments involve subjecting a product placed between two electrodes, usually immersed in an aqueous solution, to high-intensity electric fields (between 0.5 and 30 kV/cm) by intermittently applying short-duration pulses (microseconds to milliseconds). Although the application of electric fields inherently introduces energy into the system, the total treatment time is extremely brief—usually less than one second in continuous flow systems. This short exposure time ensures that the temperature increase is minimal and transient. Consequently, thermal damage is effectively negligible, preserving the physicochemical and sensory qualities of the treated food. If the electric field intensity is sufficiently high, electroporation of the cytoplasmic membrane occurs, increasing its permeability to ions and macromolecules [25]. This phenomenon can be reversible or irreversible depending on the electrical parameters applied. In reversible electroporation, the membrane reseals after the treatment, whereas in irreversible electroporation, the structural integrity of the membrane is permanently compromised. This irreversible loss of selective permeability disrupts cellular homeostasis and can lead to cell death [26].

Although PEF is widely used for the non-thermal inactivation of bacteria in food [27], its application for parasite inactivation has been limited. Recent studies targeted the inactivation of nematode larvae as *Anisakis* spp. and *Trichinella* spp. [26,28], while there is limited information on the effect of PEF on infective forms of protozoa (oocysts), except for the work by Haas and Aturaliye [29]. However, 26 years have passed since this study, and PEF technology has greatly improved, offering much greater treatment possibilities today. PEF technology has been successfully scaled up for industrial use, with continuous-flow systems processing thousands of liters per hour. It is already applied in sectors like juice pasteurization and winemaking, demonstrating its feasibility for high-throughput applications. Despite these technological developments, no systematic investigation has been conducted to assess how PEF influences protozoan oocyst inactivation. Therefore, this study addresses a significant gap in the literature by being, to our knowledge, the first to evaluate PEF-mediated permeabilization of *Cryptosporidium* oocysts, under well-controlled experimental conditions, using modern and high-performance PEF equipment.

The objective of this work is to evaluate the potential of PEF treatments to inactivate *Cryptosporidium* spp. oocysts for the decontamination of water and food matrices such as fruit juices. The aim is to achieve irreversible electroporation of *Cryptosporidium* cell membranes, thereby compromising their physiology to the extent of causing inactivation and preventing water and food-borne transmission.

## 2. Materials and Methods

### 2.1. Isolation and Purification of Cryptosporidium Oocysts

*Cryptosporidium* spp. oocysts were isolated from fecal samples collected from 10-day-old calves naturally infected within a herd located in Aragón, Spain. Fecal samples were obtained directly from the rectum, placed in sterile containers and transported to the Faculty of Veterinary Medicine at the University of Zaragoza for further analysis. These fecal specimens were collected by the herd veterinarian for diagnostic analysis of parasitic protozoa.

Oocysts in fecal samples were concentrated and purified through successive centrifugations using a sodium chloride (NaCl) flotation method. Purified oocysts were suspended in microtubes at a final concentration of 3 × 10^6^ oocysts/mL and stored at 4 °C until analysis.

### 2.2. PEF Treatment

The PEF equipment used in this study was a commercial device (Vitave, Prague, Czech Republic) capable of delivering pulses of up to 20 kV. The system applies monopolar square-wave pulses of variable width (500 ns–100 µs), with a maximum current intensity of 500 A, and allows operation at frequencies up to 50 kHz. The applied voltage was monitored using a high-voltage probe (P6015A, Tektronik, Wilsonville, OR, USA), while the current intensity was recorded with a current probe (HCT5514, Meatrol^®^Electrical, Shanghai, China). Both probes were connected to an oscilloscope (TDS 220, Tektronik Wilsonville, OR, USA).

Treatments at **room temperature**: a suspension of oocysts (0.44 mL, with a conductivity of 1 mS/cm) was introduced into a cylindrical static treatment chamber with parallel electrodes, featuring specific dimensions (gap: 0.25 cm; diameter: 1.5 cm), using a sterile 1 mL syringe (TERUMO, Leuven, Belgium) with a 20 G × 1” (0.9 × 25 mm) needle (TERUMO, Leuven, Belgium). Samples for investigating the effect of PEF on oocysts were subjected to between 20 and 90 square-wave pulses of 3 μs at different electric field intensities (15, 25, and 35 kV/cm). These treatments corresponded to specific energies ranging from 14.65 to 320.27 kJ/kg. Control and treated oocyst experiments were performed in duplicate.

**Temperature-controlled** treatments: The batch parallel electrode treatment chamber used for the temperature-controlled treatments was based on a previous design of Heinz et al. [30] and later of [31]. The treatment chamber consisted of a cylindrical polypropylene tube closed with two polished stainless steel cylinders of 1.77 cm^2^ surface and 4 cm length. The inner part of the electrodes was empty, and dielectric oil (conductivity: 1.4 µS/cm) tempered at different temperatures was recirculated through both electrodes to temper the treatment medium at different temperatures and to maintain a constant temperature in the medium during the PEF treatments. A small hole in the cylindrical plastic tube (chamber) was used to introduce and remove the microbial suspension. The distance between electrodes was 0.25 cm. Both the oocyst suspensions in water and the juices inoculated with oocysts introduced into the chamber for each treatment had a volume of 0.44 mL. Before and immediately after applying the PEF treatment, in order to know the temperature in different zones of the treatment chamber, the temperature was measured using an Infrared Thermal imaging camera (FLIR E6 PRO, Teledyne FLIR LLC, Wilsonville, OR, USA,) and a fiber-optic temperature probe (Fiso, Québec, QC, Canada).

### 2.3. Assessment of Thermal Effects on Oocyst Membrane Permeabilization in the Absence of PEF

To isolate the thermal effects from those induced by PEF and to better understand the heat resistance of oocysts, oocyst solutions were placed in the PEF treatment chamber pre-tempered at 40, 50, 60 and 70 °C for 1, 5, and 10 min, with the PEF system turned off during the thermal exposure. This approach allowed the assessment of membrane permeabilization attributable exclusively to temperature.

PEF-treated and thermal treated samples were processed immediately for microscopic observation and flow cytometry analysis in order to minimize the time lapse between treatment and evaluation.

### 2.4. Assessment of Cryptosporidium Oocyst Viability

#### 2.4.1. Microscopy

To evaluate the viability of *Cryptosporidium* oocysts and potential viability loss following different treatments, a fluorescence microscopy-based method was employed, using the differential uptake of the vital dyes 4′,6-diamidino-2-phenylindole (DAPI) and propidium iodide (PI). DAPI is a membrane-permeant fluorochrome that binds to double-stranded DNA, staining the nuclei of all sporozoites of the oocysts regardless of viability. PI, in contrast, is a membrane-impermeant dye that only penetrates oocysts with compromised membranes; it intercalates with nucleic acids and emits red fluorescence. The uptake of PI by oocysts reflects membrane permeabilization, which serves as a functional indicator of compromised integrity and, consequently, loss of viability.

Aliquots containing 3 × 10^6^ oocysts were transferred into 1.5 mL microtubes and pre-incubated in acidified Hanks’ Balanced Salt Solution (HBSS, pH 2.75) for 1 h at 37 °C to facilitate dye uptake. This was followed by two washes with HBSS (pH 7.2) to restore physiological pH. Then, 10 µL of DAPI working solution (2 mg/mL in absolute methanol) and 10 µL of PI working solution (1 mg/mL in 0.1 M phosphate-buffered saline, pH 7.2) were added to each sample and incubated for 2 h at 37 °C in the dark. To prevent DAPI crystallization, samples were washed twice with HBSS adjusted to pH 4, following the protocol described by Bukhari et al. [32].

Oocysts were visualized using an epifluorescence microscope (Nikon, Mod. L-Kc, Nippon Kogaku KK, Tokyo, Japan) equipped with a UV excitation filter (350–365 nm) for DAPI and a green excitation filter (545–546 nm) for PI. A minimum of 100 oocysts were examined per sample. Oocysts showing only blue fluorescence (DAPI-positive, PI-negative) were considered viable, whereas those exhibiting both blue and red fluorescence (DAPI-positive, PI-positive) were classified as non-viable due to membrane permeabilization. This loss of selective membrane integrity reflects compromised viability and parallels the concept of electroporation-induced damage explored in later analyses.

The data of treated oocysts were normalized to the background permeability observed in the controls. The viability percentage of the negative control oocysts (*NC*) was adjusted to 100%, and the viability percentage of the treated oocysts (*TO*) was corrected by the same factor using the following formula:

(1) $$Corrected\;TO=\left(\frac{Observed\;TO}{Observed\;NC}\right)\times 100$$

The percentage of permeabilized oocysts was calculated as the difference between 100% and the viability percentage.

#### 2.4.2. Flow Cytometry

To quantitatively assess membrane permeabilization in *Cryptosporidium* oocysts following PEF treatments, with greater sensitivity and resolution than microscopy, flow cytometry was employed as the principal analytical technique. A dual-fluorescence staining protocol was carried out using a Guava^®^ easyCyte™ flow cytometer (Luminex^®^, Tokyo, Japan), which operates with a 488 nm blue laser for excitation and detects fluorescence through specific optical filters.

*Cryptosporidium* oocysts were identified using the Aqua-Glo™ G/C Direct Fluorescent Antibody reagent (Waterborne Inc., New Orleans, LA, USA), which contains fluorescein isothiocyanate (FITC)-labeled mouse monoclonal antibodies directed against cyst and oocyst outer wall antigenic sites (epitopes) of *Giardia lamblia* and *Cryptosporidium parvum*. This reagent is genus-specific and binds exclusively to the cysts and oocysts of these two parasites. Green fluorescence from FITC-labeled oocysts was detected using a 525/30 nm bandpass filter (GRN-B), enabling specific identification of antibody-bound oocysts. Membrane permeabilization—and consequently, loss of viability—was assessed using propidium iodide (PI), whose fluorescence was detected using a 695/50 nm bandpass filter (RED-B).

Following PEF treatment, oocysts (370 µL, 10^6^ oocyst/mL) were pre-incubated at pH 2.75 for 1 h at 37 °C, washed twice with HBSS (pH 7.2), and incubated with 30 µL of PI solution (0.1 mg/mL) for 2 h at 37 °C in the dark. During the final 25 min of incubation, 4.5 µL of the antibody reagent was added to each tube. Samples were then analyzed in duplicate using the flow cytometer, with 8000 events recorded per sample at a flow rate of 0.59 µL/s. Events emitting green fluorescence above 3.0 × 10^3^ a.u. were considered FITC-positive, while those emitting red fluorescence above 3.0 × 10^3^ a.u. were considered PI-permeable. The percentage of permeabilization was calculated based on the proportion of oocysts exceeding this fluorescence threshold.

**Figure A1:**
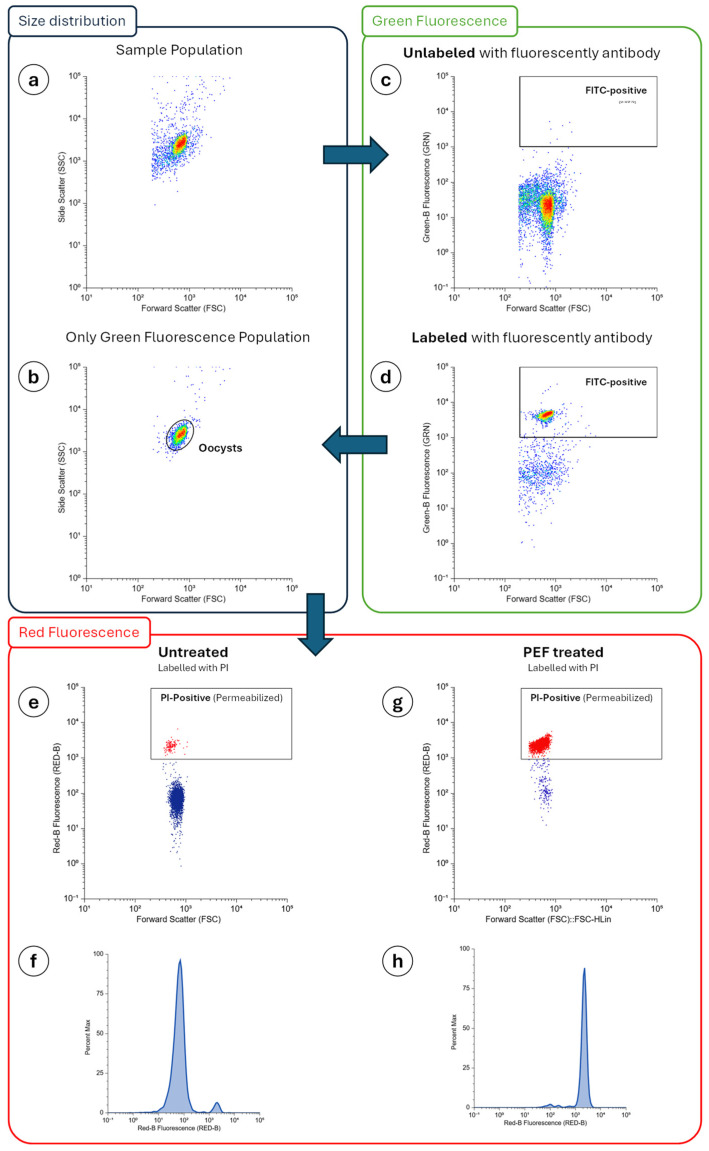
Flow cytometry gating strategy used to evaluate membrane permeabilization of *Cryptosporidium* oocysts after PEF treatment. (**a**) Initial FSC vs. SSC plot showing the total particle population in the samples. (**b**) Sub-gating of the oocyst population based on size and internal complexity (FSC vs. SSC), selecting only events exhibiting green fluorescence (FITC-positive) to ensure specific identification of antibody-labeled oocysts. (**c**,**d**) Green fluorescence (GRN-B) from samples without and with FITC-labeled antibody, respectively; only labeled samples exhibit a clear FITC-positive signal used to identify oocysts. (**e**,**g**) PI uptake in untreated and PEF-treated oocysts, respectively, measured in the red fluorescence channel (RED-B); PI-positive oocysts appear in red. (**f**,**h**) Histogram overlays representing red fluorescence intensity (RED-B) in untreated and PEF-treated oocysts, respectively, showing an increase in PI uptake after PEF treatment. Data correspond to 8000 events acquired per sample.

Data acquisition began by visualizing the total particle distribution via forward scatter (FSC) versus side scatter (SSC) (Figure A1a). To identify the oocyst population and exclude smaller contaminants with overlapping size distributions, events emitting green fluorescence (Figure A1d) were gated to isolate specifically labeled oocysts (FITC-positive) (Figure A1b). Within this gated oocyst population, red fluorescence from PI was analyzed to differentiate between permeabilized oocysts (PI-positive) and intact oocysts (PI-negative) (Figure A1e,g).

This gating strategy enabled accurate and selective quantification of PEF-induced membrane permeabilization by analyzing PI uptake specifically within the FITC-positive oocyst population, while excluding non-target particles and background debris.

### 2.5. Fruit Juices

The PEF treatments applied in the buffer were validated with the oocysts suspended in two fruit juice matrices. Commercial pasteurized 100% fruit juice made from Golden and Granny Smith apples was used. Carrot juice was prepared using the Juissen 2 cold press juicer (Sojamatic, Barcelona, Spain) and commercial carrots. The physicochemical properties of the juices are shown in Table 1. The juices were inoculated with 2 × 10⁶ oocysts/mL and treated by PEF at 25 kV/cm (30 pulses of 3 µs) applied at different temperatures (20, 30, and 40 °C).

### 2.6. Experimental Design

Response surface methodology (RSM) was used to evaluate the effect of electric field strength (15–35 kV/cm), treatment time (60–180 μs), and input energy (14.65 to 320.27 kJ/kg) on the permeabilization of *Cryptosporidium* to PI. The data obtained were modeled with the following second-order polynomial equation:

(2) $$Y=\beta_{0}+{\Sigma_{i=1}}^{k}\;\beta_{i}X_{i}+{\Sigma_{i=1}}^{k}\;\beta_{ii}{X_{i}}^{2}+\Sigma_{i}{{<\;}_{j}}^{k}\;\beta_{ij}X_{i}X_{j}$$

In which *Y* is the response variable to be modeled, *X_i_* and *X_j_* are independent factors, *β*_0_ is the intercept, *β_i_* represents the linear coefficients, *β_ii_* represents the quadratic coefficients, *β_ij_* represents the cross-product coefficients, and k is the total number of independent factors. A backward regression procedure was used to determine the models’ parameters. This procedure systematically removed the effects that were not significantly associated (*p* > 0.05) with the response until a model with a significant effect was obtained.

### 2.7. Statistical Analysis

Experiments were performed in triplicate, and the presented results are the mean ± 95% confidence interval. One-way analysis of variance (ANOVA) using Tukey’s test was performed to evaluate the significance of differences between the mean values. The differences were considered significant at *p* < 0.05. A multiple regression analysis was conducted for fitting Equation (2) to the experimental data and significant terms of the model were determined by ANOVA. The central composite design and the corresponding data analysis were carried out by using the software package Design-Expert 13 (Stat-Ease Inc., Minneapolis, MN, USA).

## 3. Results and Discussion

### 3.1. Influence of the Electric Parameters on Cryptosporidium Oocysts Permeabilization to PI

#### 3.1.1. Effect of Electric Field Strength and Treatment Time

The influence of electric field strength and treatment time (number of pulses × pulse width) on the permeabilization of *Cryptosporidium* to PI was investigated in order to find the optimal PEF treatment conditions that maximize inactivation. The results confirm that a certain level of permeabilization occurs after PEF treatments, with a clear direct effect of electric field strength and treatment duration. The range of permeabilized oocysts varied between 2.09% and 96.11%, depending on the treatment parameters, highlighting the importance of fine-tuning the electrical conditions to achieve effective membrane disruption.

#### 3.1.2. Regression Modeling and Statistical Significance

In order to quantify the influence of electric field strength and treatment time on the permeabilization of *Cryptosporidium*, a multiple regression analysis was performed, fitting the experimental data to Equation (2). The analysis resulted in the following equation after removing non-significant terms (*p* > 0.05):

(3) $$\%\;Permeabilized\;Cryptosporidium\;oocyst=72.707-7.571E-0.076t+0.009Et+0.194E^{2}\;$$

Results of the analysis of variance (ANOVA) are shown in Table 1, including the statistical measures used to assess the adequacy of the generated models. The Model F-value of 101.82 implies the model is significant. There is only a 0.01% chance that an F-value this large could occur due to noise. *p*-values less than 0.05 indicate model terms are significant. In this case A, B, AB, and A^2^ are significant model terms. The lack of fit F-value of 0.77 implies the lack of fit is not significant relative to the pure error. There is a 61.93% chance that a lack of fit F-value this large could occur due to noise. Non-significant lack of fit is good; we want the model to fit.

The F-values of the model parameters displayed in Table 2 are indicators of the significance of the variables’ effects. According to those F values, the electric field strength’s linear term (F = 304.45), along with the electric field strength’s quadratic (F = 39.64) were the two most significant variables, indicating that changes in these factors had the most influence on the oocysts permeabilization. The linear term of treatment time (F = 16.84) and the interaction between electric field and treatment time (F = 4.30) were also significant, but had lower F-values. The presence of that interaction term means that the effect of electric field strength on oocyst permeabilization slightly depends on the treatment time. The fit statistics of the model developed are detailed in Table 3.

The model showed a strong goodness-of-fit, with a coefficient of determination (R^2^) of 0.899, indicating that approximately 89.9% of the variability in the response variable was explained by the fitted model. The adjusted R^2^ (0.890) was close to the R^2^ value, suggesting that the model was not overfitted and that the included predictors are relevant. Furthermore, the predicted R^2^ (0.877) demonstrated a good agreement with both R^2^ and adjusted R^2^, confirming the model’s predictive capability and its robustness in estimating responses for new observations.

#### 3.1.3. Interpretation of the Model and Response Surface Visualization

**Figure 1:**
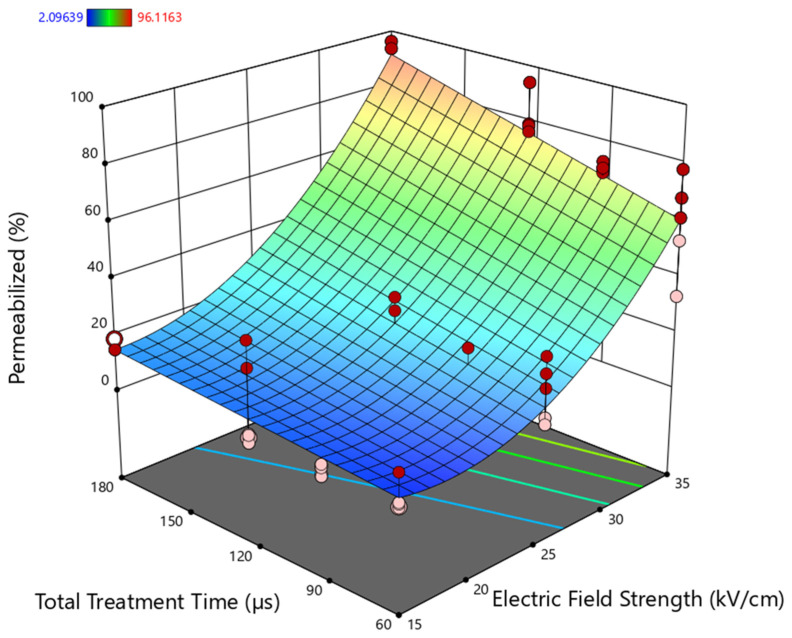
Three-dimensional surface plot illustrating the effects of electric field strength and treatment time on the PI permeabilization percentage of *Cryptosporidium* oocysts after Pulsed Electric Field (PEF) treatments. The surface represents the model prediction, with a color gradient indicating the magnitude of the response variable: blue for lower values and red for higher values. The color bar at the top-left corner corresponds to the predicted response values, ranging from 2.00689 (blue) to 96.1163 (red). Red dots represent actual experimental values that are higher than the model predictions, while pink dots indicate values that are lower than predicted.

In order to illustrate the influence of electric field strength and treatment time on the permeabilization of *Cryptosporidium* oocyst membrane, a graphical representation (Figure 1) has been generated using the regression model (Equation (3)) considering the responses within the range of experimental conditions assayed.

As shown in the three-dimensional response surface, a progressive increase in permeabilization is observed with increasing both parameters. However, the contribution of each factor differs significantly. Electric field strength exhibits a non-linear, quadratic effect, evidenced by the pronounced curvature along the E-axis, suggesting a threshold behavior consistent with membrane electroporation theory [25]. At subcritical field levels < 25 kV/cm, the increase in membrane permeability is limited, but it escalates rapidly once the threshold is exceeded. A similar quadratic dependence of electric field strength has been reported in other parasites; for example, Abad et al. [28] described that the inactivation of *Anisakis* larvae followed a non-linear pattern with respect to electric field intensity, where moderate increases in the field resulted in significant higher lethality. Conversely, the effect of treatment time is linear and comparatively modest, indicating that prolonged exposure alone cannot compensate for subthreshold field intensities. This reinforces the principle that sufficient transmembrane potential, which is primarily governed by field strength, is essential for effective pore formation [33].

Furthermore, a synergistic interaction between electric field intensity and pulse duration is evident, as reflected by the positive coefficient of the E·t interaction term in the regression model. This synergy implies that simultaneous increases in both parameters produce a greater-than-additive effect on oocyst permeabilization. The presence of experimental data points both above and below the modeled surface highlights natural biological variability and potential experimental factors not captured by the model, such as oocyst age, aggregation, or microenvironmental conditions.

According to the model, electric field strengths higher than 30 kV/cm are required to achieve more than 50% permeabilization of *Cryptosporidium* oocysts. For instance, applying treatments at room temperature with 30 kV/cm and 180 µs resulted in 55.1% permeabilization of the oocysts. In contrast, more intense conditions (35 kV/cm and 180 µs) led to nearly 90% permeabilization of *Cryptosporidium* oocysts. The intensity of the PEF parameters (electric field and treatment time) required to achieve permeabilization highlights the strong resistance of *Cryptosporidium* oocysts to electroporation.

The only previous study of PEF on Cryptosporidium, conducted by Haas and Aturaliye [29], reported limited effectiveness of electroporation alone for oocyst inactivation, with only minimal effects observed even at energy levels up to 386 kJ/kg. However, the authors reported only that the maximum voltage used in the experiments was 3 kV; they did not directly report the electric field strength in kilovolts per centimeter (kV/cm). The electrode gap (distance between electrodes in the cuvette) is not explicitly stated in the paper, so we cannot calculate the electric field. If we assume they used a standard 0.2 cm (2 mm) cuvette, which is common in electroporation setups and coincides with the volume they used per sample, the maximum field strengths would be 3 kV/0.2 cm = 15 kV/cm. As demonstrated in the present study, the applied electric field used by Haas and Aturaliye [29] remained well below the threshold required to electroporate the resistant forms of Cryptosporidium. Their results indicated that electroporation was insufficient as a standalone inactivation method but still enhanced the efficacy of chemical disinfectants such as combined chlorine and hydrogen peroxide through synergistic interactions. These discrepancies in the efficacy of PEF may be attributed to differences in the technological development of PEF equipment and treatment parameters (e.g., electric field strength, pulse duration). While Haas and Aturaliye [29] applied up to 20 pulses from 0 to 99 µs, they did not specify the exact treatment time. The present study implements a comprehensive and well-defined experimental design, applying 20 to 60 pulses of 3 µs each, with precise control over electric field intensity (15–35 kV/cm) and application temperature. This setup enables accurate monitoring of the actual voltage and current applied, thanks to the probes connected to the equipment. Furthermore, viability assessment in the current work was supported by flow cytometry using genus-specific antibodies, providing higher resolution than the excystation-based methods used in 1999. Collectively, these findings suggest that advancements in PEF technology and methodology have significantly improved its standalone potential for Cryptosporidium inactivation.

### 3.2. Influence of the PEF Treatment Temperature on the Cryptosporidium Oocysts Permeabilization to PI

#### 3.2.1. Evaluation of Thermal Resistance of Oocysts in the Absence of PEF

After demonstrating that PEF could be an effective procedure for *Cryptosporidium* oocyst permeabilization, but considering their high resistance and the intense treatment parameters required, the effect of applying the treatments at temperatures above room temperature was investigated to determine whether it could enhance the effectiveness of PEF and facilitate its implementation.

**Figure 2:**
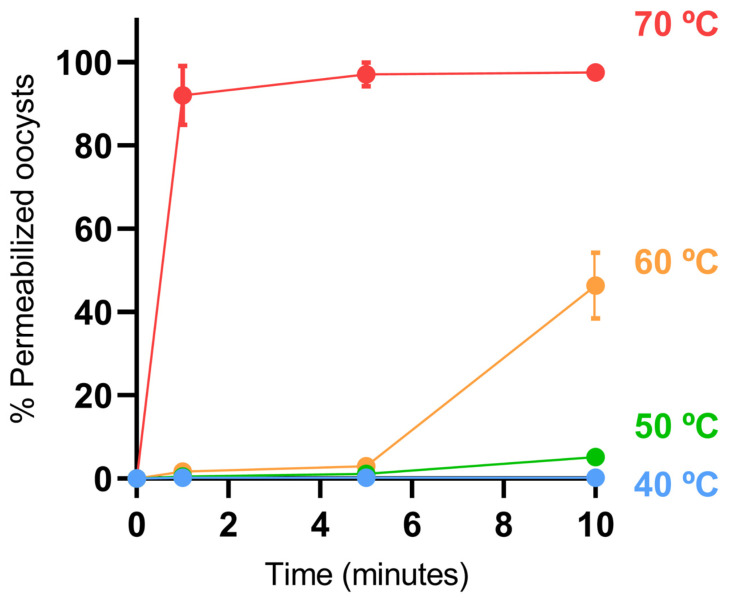
Thermal resistance of *Cryptosporidium* oocysts in buffer: percentage of permeabilized oocysts after exposure to 40 (blue), 50 (green), 60 °C (orange) and 70 °C (red) for 1, 5, and 10 min.

Before selecting the temperatures for the application of PEF treatments, a study was conducted to evaluate the thermal resistance of isolated *Cryptosporidium* oocysts. The aim was to rule out a purely thermal effect and to identify moderate temperatures that could exert a synergistic effect with PEF treatments without affecting oocyst viability on their own. Figure 2 shows the percentage of *Cryptosporidium* oocysts permeabilized to PI after different exposure times (1, 5, and 10 min) at four temperatures (40, 50, 60, and 70 °C). Permeabilization was negligible in all cases, except at the highest temperature (70 °C) for all the times assayed and after the longest exposure time (10 min) at 60 °C. Therefore, it can be said that temperatures below 60 °C are ineffective for inactivating *Cryptosporidium* oocysts, even after prolonged exposure times (5 min).

Studies on the permeabilization of *Cryptosporidium* oocysts to PI after heat treatment were commonly conducted to assess post-treatment viability. PI is a fluorochrome that does not penetrate intact cell membranes, so its uptake by oocysts indicates damage to the membrane, presumably associated with a loss of viability. This staining gained popularity due to its simplicity and rapid results [34,35,36]. Campbell et al. [34] reported a highly significant correlation (r = 0.997) between a dual-staining assay using DAPI and PI and in vitro excystation, suggesting that PI staining can reliably indicate oocyst viability under certain conditions. However, comparisons with other methods suggest that PI staining tends to overestimate viability compared to infectivity assays because some non-infectious oocysts may still exclude the dye and be misclassified as viable. Additionally, SYTO-17 staining, a fluorogenic nucleic acid dye method, has demonstrated high sensitivity (~96%) and specificity in distinguishing heat-killed from viable oocysts. Like other dye-based methods, it still relies on the assumption that membrane integrity equates to infectivity [37]. More recent approaches include RT-qPCR, PMA-qPCR, and RNA FISH, which aim to detect metabolic activity or intact nucleic acids associated with viable oocysts. For instance, PMA-qPCR selectively amplifies DNA only from oocysts with intact membranes, improving specificity. Meanwhile, RNA FISH targets ribosomal RNA to identify viable oocysts but its reliability can be affected by RNA degradation in environmental matrices [36]. All of these molecular methods focus on the integrity of a specific part of the oocyst, such as the membrane, or on certain metabolic activities, but they do not directly measure its infective capacity, which—aside from cell culture assays—would require confirmation using animal models. However, as animal models (e.g., mice) are increasingly avoided due to ethical concerns, and loss of membrane integrity correlates well with infectivity, PI staining remains, in our view, a valid and practical approach for preliminary infectivity assessments.

The results obtained in the present study are consistent with previous publications on the inactivation of *Cryptosporidium* oocysts that include thermal treatments (temperature-time combinations) within the temperature range studied. In a pivotal study by Fayer [38], oocysts suspended in water were subjected to controlled heating and infectivity was evaluated through bioassays in neonatal BALB/c mice, followed by histological examination of intestinal tissues. The results demonstrated that oocysts remained infectious when water temperatures reached up to 67.5 °C within 1 min. However, complete inactivation was achieved when oocysts were exposed to temperatures of 72.4 °C. These findings in animal models are consistent with our results with PI uptake and reinforce the notion that moderate heat treatments below 60 °C are ineffective. Importantly, this study, as in our case, eliminated confounding factors such as food matrices or organic matter, thereby providing robust data on heat inactivation in clean aqueous suspensions. Nevertheless, the authors noted that results might differ in more complex matrices like milk, emphasizing the need for matrix-specific validation. Similarly, the study of Jenkins et al. [35] on the thermal inactivation of *C. parvum* oocysts using differential uptake of DAPI and PI demonstrated that exposure to increasing temperatures accelerated the shift in oocyst membrane permeability from DAPI^−^PI^−^ (impermeable, viable) to DAPI^+^PI^+^ (permeable, non-viable). Nine minutes at 70 °C was required to achieve 75% DAPI^+^PI^+^ oocysts, while 25% were still considered potentially viable. In contrast, 2 min of exposure at 80 °C resulted in complete transition to DAPI^+^PI^+^, indicating total inactivation. In our study, we used a fluorescein-labeled antibody that specifically binds to the wall of *Cryptosporidium* oocysts. Therefore, after parameter selection, gaining, and gating, we ensured that the events analyzed by flow cytometry corresponded to oocysts, making our assessment of PI uptake potentially more accurate than in older studies where DAPI and microscopy was used. Temesgen et al. (2021) [39] employed a novel approach to assess the viability of *C. parvum* oocysts following thermal inactivation treatments by developing a reverse transcription quantitative PCR (RT-qPCR) method based on the expression of genes induced by oxidative stress. This technique is grounded in the principle that only metabolically active (i.e., viable) oocysts respond to oxidative stress by upregulating specific genes, such as thioredoxin and COWP7. Their results indicated that oocysts exposed to 60 °C for 2 min still exhibited measurable expression of these stress-responsive genes, suggesting a lack of inactivation, whereas heating at 80 °C for 3 min was required to achieve complete inactivation. This study, which evaluates metabolic viability rather than PI uptake, is also consistent with the findings of the present work. Therefore, it is confirmed that temperatures below 60 °C should not have an effect on *Cryptosporidium* inactivation on their own, and thus temperatures below this threshold (25–60 °C) were selected for the experiments evaluating the influence of application temperature during PEF treatments.

#### 3.2.2. Synergistic Effect of Temperature and PEF

The PEF treatment conditions selected for this experiment (25 kV/cm, 90 µs) were chosen based on the results obtained from the response surface model described earlier. Specifically, the model revealed a threshold electric field around 25 kV/cm, below which oocyst permeabilization was minimal. To remain at this threshold level and avoid inducing high baseline permeabilization, which could mask potential synergistic effects between PEF and temperature, 25 kV/cm was selected as the field intensity. Additionally, as the model indicated that treatment time had a relatively minor effect compared to field strength, a moderate duration of 90 µs was employed. This exposure was sufficient to induce approximately 20–30% membrane permeabilization at room temperature, thereby creating a dynamic window to evaluate potential enhancements when combined with sublethal thermal stress.

**Figure 3:**
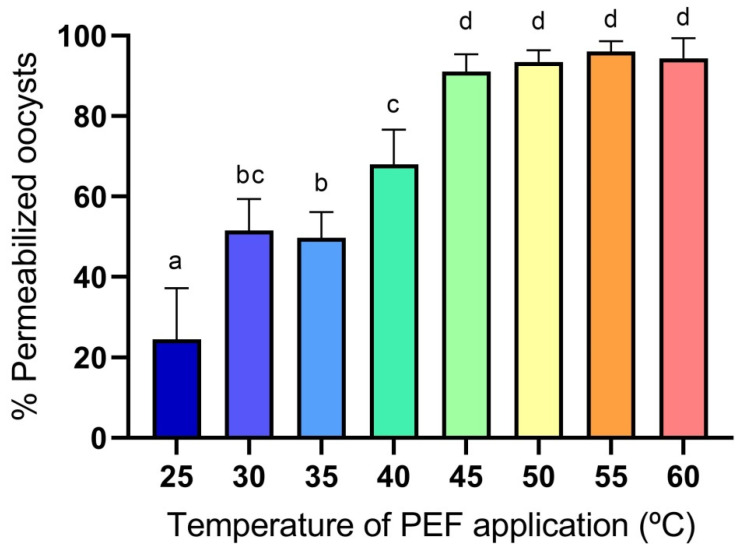
Influence of the temperature of application of PEF treatment (25 kV/cm, 90 µs) on the *Cryptosporidium* oocysts’ permeabilization to PI. Different letters indicate significant differences (*p* ≤ 0.05).

The influence of the temperature of application of PEF treatment (25 kV/cm, 90 µs) on the *Cryptosporidium* oocysts permeabilization to PI is shown in Figure 3. A significant temperature-dependent increase in oocyst permeabilization is observed when applying PEF treatments. At 25 °C, the permeabilization percentage is relatively low (~30%) with high variability. A progressive increase in temperature from 30 °C to 45 °C results in a steady increase in permeabilization, reaching approximately 85% at 45 °C. From 50 °C onwards, the effect appears to plateau, with values consistently around or above 90%, suggesting that a maximum permeabilization effect is achieved, or even that the oocysts are so severely damaged that they release their genetic material, preventing PI from binding and producing a signal. Error bars indicate that variability decreases as temperature increases, especially beyond 45 °C. These results indicate that increasing the temperature of PEF application significantly enhances oocyst permeabilization, with temperatures ≥ 40 °C maximizing the effect. This suggests a synergistic interaction between thermal energy and electric pulses, improving the efficacy of PEF treatment on the oocysts’ inactivation.

Consistent with our findings, multiple studies have examined the effects of PEF treatments on microbial inactivation—including yeasts and bacteria—at different temperatures, emphasizing the synergistic benefits of combining PEF with moderate heat. Montanari et al. [40] explored the inactivation of the yeast *Saccharomyces* (*S.*) *cerevisiae* using PEF combined with mild heat (50 °C). Preheating the yeast suspension enhanced the efficacy of PEF treatments, leading to greater microbial reductions. The combination of heat and PEF was more effective than either treatment alone. Yan et al. [41] examined the inactivation of *S. cerevisiae, Escherichia coli*, and *Bacillus* (*B.*) *velezensis* under different initial temperatures (24 °C, 30 °C, 40 °C, and 50 °C). The results indicated that increasing the temperature reduced the critical electric field intensity required for microbial inactivation. This suggests that temperature enhances the electroporation process, leading to more effective microbial inactivation. Notably, *B. velezensis*, known for its spore-forming capability, showed increased resistance, but the combination of higher temperatures with PEF treatments significantly improved inactivation rates. In this sense, similarly to bacterial spores, the oocysts of *Cryptosporidium* exhibit remarkable structural adaptations that confer resistance to environmental stresses, ensuring their survival in adverse conditions. Bacterial endospores, such as those formed by *Bacillus* and *Clostridium* species, are characterized by a multilayered structure comprising an exosporium, spore coat, cortex, and core. The spore coat, rich in proteins, provides chemical and enzymatic resistance, while the cortex, composed of peptidoglycan, aids in maintaining dehydration of the core. The core contains DNA stabilized by small acid-soluble spore proteins (SASPs) and high concentrations of calcium dipicolinate, contributing to resistance against heat, radiation, and desiccation [42]. Similarly, *Cryptosporidium* oocysts possess a robust wall structure with inner and outer layers composed of proteins and carbohydrates. This wall confers resistance to chemical disinfectants and environmental pressures. The oocyst wall’s unique composition, including acid-fast lipids, contributes to its durability [43]. Both structures (bacterial spores and parasitic oocysts) are metabolically dormant, enabling them to withstand unfavorable conditions and persist in the environment. Their resilience poses significant challenges in public health, particularly in water treatment and food sterilization processes, necessitating advanced methods for effective inactivation.

As mentioned in the introduction, some recent research has explored the application of PEF for inactivating parasites such as *Anisakis* spp. and *Trichinella* spp. [26,28]. However, to the best of our knowledge, the influence of application temperature during PEF treatments on parasite inactivation had not been studied to date. Previous studies about permeabilization of bacteria and yeast, together with the results of the present investigation, underscore the importance of combination with mild temperature in enhancing the effectiveness of PEF treatments for microbial inactivation.

#### 3.2.3. Technological Implications of Thermally-Assisted PEF Treatments

Our study demonstrated that PEF treatments (25 kV/cm, 90 µs) applied at 45 °C induce substantial membrane permeabilization in *Cryptosporidium* oocysts, achieving up to 90% PI uptake. This high level of permeabilization is indicative of severe membrane compromise and likely loss of viability. Importantly, these effects were achieved at a field intensity notably lower than what would be required to reach similar permeabilization levels at room temperature (35 kV/cm, 180 µs). This highlights a critical benefit of thermally assisted PEF: by leveraging moderate thermal energy, it is possible to reduce the electric field intensity necessary to disrupt oocyst membranes effectively.

From a technological and industrial perspective, this finding is highly relevant. Operating at lower field intensities reduces the demand for high-voltage equipment, making PEF systems more energy-efficient, economically viable, and scalable for large-volume applications. High-voltage generators required to produce fields above 30–35 kV/cm are often expensive and difficult to implement in continuous industrial processes, whereas fields below 25 kV/cm can be achieved more easily with commercially available PEF systems, facilitating integration into existing processing lines. Therefore, combining PEF with moderate heating presents a practical strategy to enhance oocyst inactivation while minimizing infrastructure complexity.

In this context, it is worth comparing PEF with other non-thermal or minimally thermal technologies investigated for *Cryptosporidium* inactivation. UV irradiation, particularly using medium-pressure lamps, has been investigated for the inactivation of *C. parvum*. Clancy et al. [44] demonstrated that a dose of 3 mJ/cm^2^ achieved over a 3-log reduction in infectivity when applied to thin liquid layers, as assessed using neonatal mouse models. Similarly, Craik et al. [45] confirmed that UV doses of 10–25 mJ/cm^2^ could achieve 2–3 log reductions. More recent studies using UV-LEDs [46] indicated that wavelengths around 284–289 nm were particularly effective, achieving 2-log reductions in less than 40 min of exposure using in vitro assays. However, although these results may suggest that UV could be an effective disinfection strategy, its practical application is severely limited by matrix turbidity and poor penetration depth, both of which invalidate its efficacy under real-world conditions. Similarly, high hydrostatic pressure (HHP) has been assayed for inactivating resilient *C. parvum* oocysts. Slifko et al. [24] reported that HHP at 550 MPa for 60 s eliminated *C. parvum* infectivity in fruit juices, achieving over 3.4-log reductions. Similarly, Collins et al. [47] observed up to a 93.3% reduction in infectivity in oyster tissues at 550 MPa for 180 s using neonatal mouse bioassays. While highly effective, the requirement for specialized high-pressure equipment—and particularly the significant challenges related to designing systems capable of continuous treatment—prevents its practical implementation, making it another unfeasible alternative to liquid treatments. Pulsed light (PL) has also been studied, with the aim of inactivating *Cryptosporidium* oocysts in both water and food matrices through both laboratory and field-scale setups. Huffman et al. [48] reported that a point-of-use PL device achieved >4 log_10_ inactivation of *C. parvum* in water using cell culture and animal infectivity assays, highlighting its potential for household water purification systems with very low flow. On produce, PL has shown variable efficacy depending on the commodity. Craighead et al. [23] achieved up to 4.3 log_10_ reduction in oocyst infectivity on mesclun lettuce and between 2.2–2.5 log_10_ on spinach and tomatoes after 45–90 s of treatment with minimal impact on produce quality. However, despite its proven antimicrobial efficacy on surfaces, PL is not scalable for industrial food and water treatment applications because of its limited penetration depth, which, similarly to UV-irradiation, restricts inactivation to surface-level contaminants. Anecdotally, cold atmospheric plasma (CP) has shown some positive results in inactivating *Cryptosporidium* oocysts on fresh produce, such as cilantro, achieving up to a 2.03-log_10_ reduction after 180 s of exposure. However, treatment durations longer than 30 s caused visible damage to the cilantro leaves, such as drying and darkening, which may compromise sensory and nutritional quality, and standardization of the treatments was impossible due to different factors [49]. Other studies have explored the use of ultrasounds as a non-thermal method to inactivate *C. parvum* oocysts in water. Olvera et al. [50] investigated the effect of a 1 MHz ultrasound on *C. parvum* oocysts and found that continuous irradiation for 20 min led to significant inactivation, with an estimated 102.7 oocysts killed per second and minimal energy consumption. Similarly, Oyane et al. [51] utilized three different sonicators employing the squeeze-film effect and observed that a cylindrical sonicator operating at 26.6 kHz and 30 W achieved a 97% inactivation rate of oocysts at a flow rate of 33 mL/min, corresponding to a residence time of approximately 5.2 min. These findings suggested that ultrasound treatment can effectively reduce *C. parvum* viability, with efficacy influenced by factors such as frequency, power, and exposure time. However, these ultrasound-based disinfection methods require long treatment times, are very energy-demanding, particularly at higher frequencies where cavitation intensity is reduced, and most importantly are nowadays impossible to scale-up for continuous treatment.

In contrast, PEF technology offers a combination of effectiveness, energy efficiency, and scalability. Treatments are extremely rapid, compatible with continuous-flow systems, and—when combined with moderate heating—can achieve substantial inactivation of resilient pathogens like *Cryptosporidium* using lower electric fields. This positions PEF as a competitive and industrially viable alternative among current emerging disinfection technologies.

### 3.3. Validation of PEF Treatments Applied at Different Temperatures for the Inactivation of Cryptosporidium Oocysts in Carrot Juice and Apple Juice

#### 3.3.1. Effect of PEF Temperature on Oocysts Permeabilization in Juices

Several documented outbreaks of *Cryptosporidium* have been linked to the consumption of contaminated apple juice. One notable incident occurred in 1999 in the United States, where unpasteurized apple cider caused an outbreak affecting dozens of individuals, including children. Investigations revealed that the juice had been contaminated with *Cryptosporidium* oocysts, likely due to poor hygienic practices during production or the use of contaminated water [15,52] Similarly, *Cryptosporidium* oocysts have been detected on a variety of fresh produce, including carrots, lettuce, spinach, and other leafy greens, raising public health concerns due to their potential for foodborne transmission [53]. Contamination typically occurs through contact with fecally contaminated irrigation water, manure-based fertilizers, or during handling and processing. Carrots, in particular, may be at risk due to their close contact with soil, especially when consumed raw or minimally processed. The robust wall structure of the oocysts allows them to adhere to irregular surfaces and resist standard washing procedures [17] Furthermore, consumer demand for non- or minimally processed fruit juices is rising due to health awareness, preference for clean-label products, and interest in natural nutrient retention. Market projections show strong growth, with sales expected to nearly double [54]. The growing demand for minimally processed juices, combined with past outbreaks, raises significant safety concerns and highlights the need for effective non-thermal disinfection technologies. This is particularly critical given that *Cryptosporidium* oocysts are highly resistant to conventional chlorination of water used to wash fruits and vegetables and can remain viable in the acidic and refrigerated conditions typical of fruit juices.

**Figure 4:**
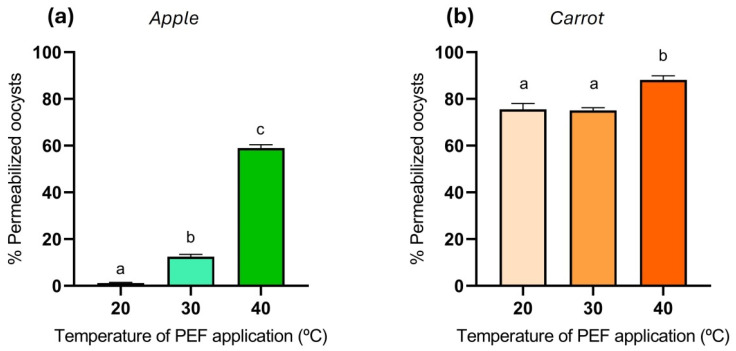
Influence of the temperature of application of PEF treatment (25 kV/cm, 90 µs) on the *Cryptosporidium* oocysts permeabilization to PI in (**a**) apple and (**b**) carrot juices. Different letters indicate significant differences (*p* ≤ 0.05).

In both apple and carrot juice, heat treatment alone up to 40 °C did not lead to statistically significant differences in oocyst permeabilization compared to the untreated control, even when the incubation at 40 °C was extended beyond one hour. The influence of the temperature of a PEF treatment application (25 kV/cm, 90 µs) in apple juice and carrot juice on the *Cryptosporidium* oocysts permeabilization is detailed in Figure 4. As shown in Figure 4a, when the PEF treatments were performed in apple juice, the percentage of permeabilized oocysts increased significantly with temperature. At 20 °C, permeabilization was minimal (<5%), while at 30 °C it reached approximately 15%. A marked increase was observed at 40 °C, with over 60% of oocysts permeabilized. In agreement with the results obtained with oocysts suspended in the buffer, these results suggest that higher application temperatures enhance the efficacy of PEF in disrupting oocyst membranes, likely due to synergistic effects between electric pulses and thermal stress. However, the lower permeabilization observed compared to buffer treatments suggests that the organic matter in the juice may exert a protective effect on the oocysts. On the other hand, Figure 4b shows the influence of PEF application temperature in carrot juice. The percentage of permeabilized oocysts remained high across all tested temperatures, with values around 75% at 20 °C and 30 °C, and slightly increasing to approximately 85% at 40 °C. These results indicate that PEF treatments were much more effective at permeabilizing oocyst membranes in this matrix in comparison to apple juice. As considerable permeabilization had already occurred at 20 and 30 °C, the improvement in permeabilization at the higher temperature was more modest.

#### 3.3.2. Influence of Matrix Properties on PEF Efficacy

Although the PEF treatments applied to apple and carrot juices were equivalent in terms of electric field strength, pulse number, and pulse width, the energy delivered to the system differed substantially due to the variation in electrical conductivity between the two juices. Carrot juice showed higher conductivity due to its natural content of minerals, organic acids, amino acids, and vitamins, which increase ionic strength and facilitate greater current flow during PEF treatment, leading to higher energy input under the same electric field conditions. The conductivity of apple juice was 1.6 mS/cm, whereas carrot juice exhibited a higher conductivity of 4.3 mS/cm. Consequently, the specific energy inputs for apple juice were 106, 143, and 164 kJ/kg at 20, 30, and 40 °C, respectively, while those for carrot juice were higher, reaching 235, 248, and 262 kJ/kg at the same temperatures. The greater energy input during the treatments applied to carrot juice likely accounted for the higher level of oocyst permeabilization observed in this matrix compared to apple juice. The energy input applied during PEF treatments plays a critical role in the inactivation of microorganisms. Higher energy inputs generally lead to increased membrane permeabilization, which enhances microbial inactivation by facilitating irreversible electroporation and loss of cellular integrity. However, the extent of inactivation also depends on factors such as microbial type, medium conductivity, and temperature. Several studies have demonstrated that increasing the specific energy input improves the effectiveness of PEF in reducing microbial loads in liquid foods [55,56]. Specifically in parasites, Abad et al. [28] demonstrated that increasing the specific energy input significantly enhanced the efficacy of PEF treatments in inactivating *Anisakis* larvae.

Contrary to expectations, the treatments were more effective in the neutral-pH juice (carrot juice, pH 5.94) compared to the acidic juice (apple juice, pH 3.40). This may be explained by the higher energy applied during the PEF treatments in carrot juice, which could have masked any potential synergistic effect of acidic pH on oocyst permeabilization in the apple juice matrix. In the case of bacterial and yeast inactivation, the pH of the treatment medium significantly influences the efficacy of PEF treatments. Several studies have demonstrated that acidic environments increase microbial susceptibility to PEF. For example, Jin et al. [57] showed that reducing the pH from 7.0 to 4.0 during PEF treatment led to a substantial decrease in *E. coli* counts in liquid whole egg, indicating a synergistic interaction between low pH and PEF. Likewise, Li et al. [58] reported that *S. cerevisiae* exhibited higher inactivation rates at pH 4.0 than at pH 7.0 under PEF, suggesting that acidic conditions may weaken membrane integrity and enhance electroporation. These findings highlight the relevance of pH in optimizing PEF-based microbial inactivation strategies. However, since the treatments in our study differ significantly in terms of energy input, no conclusions can be drawn regarding the specific effect of matrix pH on the permeabilization of oocysts following PEF treatments at different application temperatures.

In addition to the pH of the medium, the efficacy of inactivation treatments for *Cryptosporidium* oocysts is strongly influenced by the surrounding matrix. Studies have shown that organic-rich environments—such as food matrices or manure—can exert protective effects on oocysts, thereby reducing the effectiveness of treatments compared to simpler systems like buffer solutions. For instance, Petersen et al. [59] observed that the inactivation rate of *C. parvum* oocysts was notably lower in cattle slurry compared to distilled water when treated with ammonia, suggesting that components in the slurry may shield oocysts from chemical agents. Similarly, Temesgen et al. [39] highlighted that standard inactivation methods effective in water might not yield the same results in food matrices, emphasizing the need to consider matrix composition in treatment strategies. Natural juice components—such as total polyphenols, polysaccharides, and vitamins—may influence the effectiveness of PEF treatment by either protecting or enhancing the inactivation of *Cryptosporidium* oocysts. Our results, in agreement with the literature, underscore the importance of tailoring inactivation approaches to specific environmental contexts and food matrices to ensure the effective control of *Cryptosporidium* oocysts.

#### 3.3.3. Industrial Feasibility, Regulatory Considerations and Limitations

Several studies have demonstrated effective microbial inactivation, particularly of bacteria, in both apple and carrot juices using PEF, with no significant alterations in color, flavor, or nutritional composition. The minimal impact on organoleptic and nutritional properties positions PEF as a promising non-thermal alternative to conventional pasteurization, especially in industrial settings where product quality and shelf-life extension are critical [60,61]. However, despite its strong potential, the industrial adoption of PEF for juice pasteurization remains limited. This is largely due to the high capital investment required for equipment, the complexity of process validation for different juice matrices, and the absence of standardized regulatory guidelines for microbial safety. Consequently, PEF is currently applied primarily in niche markets focused on premium juice products, where quality preservation justifies the associated costs.

Regulatory guidelines for microbial safety in foods typically focus on bacterial pathogens, while protozoan parasites such as *Cryptosporidium* remain less specifically addressed, especially in fruit and vegetable juices. Although the oocysts are highly resistant to conventional disinfection methods, including chlorination, and have been associated with foodborne outbreaks related to fresh produce and beverages, current regulations—such as the FDA’s HACCP guidance—acknowledge *C. parvum* as a relevant pathogen but do not establish explicit microbial limits for its presence in juices [62]. Similarly, no specific regulatory thresholds have been defined by other major authorities as the European Food Safety Authority [63]. This regulatory gap poses a significant challenge for the juice industry, as oocysts can persist in minimally processed products without altering their organoleptic properties, making contamination difficult to detect and consequently, posing a risk for public health. In this context, non-thermal processing technologies such as PEF offer a promising industrial strategy for achieving oocyst inactivation while preserving the sensory and nutritional quality of the final product.

However, despite the potential of PEF, some limitations of the present study should be acknowledged. The assessment of oocyst inactivation was based on PI uptake as a proxy for membrane permeabilization, which does not directly confirm the loss of infectivity. In vivo assays should be performed to validate this outcome. Furthermore, the experiments were conducted under controlled laboratory conditions using model matrices, and although apple and carrot juices are relevant systems, the variability in industrial formulations and processing environments may affect treatment reproducibility. Future studies should address these limitations to strengthen the robustness and scalability of PEF as a disinfection strategy.

## 4. Conclusions

This study demonstrates that Pulsed Electric Field (PEF) technology can effectively permeabilize *Cryptosporidium* oocysts suspended in water, apple juice, and carrot juice, particularly when treatments are applied at moderate temperatures (>40 °C). The findings highlight the critical role of electric field strength, treatment time, and application temperature in achieving high levels of oocyst membrane disruption, with permeabilization exceeding 90% under optimized conditions. Notably, the surrounding matrix influenced treatment efficacy, with higher inactivation observed in carrot juice—likely due to greater conductivity and thus energy input—compared to apple juice. These results support the potential of PEF as a non-thermal alternative for enhancing the microbiological safety of minimally processed fruit juices, with minimal impact on organoleptic quality. Given the growing demand for fresh-like beverages and the resilience of *Cryptosporidium* to conventional sanitizers, PEF offers a scalable, energy-efficient solution for industrial applications targeting protozoan pathogens. Further studies incorporating infectivity assays are recommended to fully validate oocyst inactivation and guide regulatory acceptance.

## Figures and Tables

**Table 1:** Physicochemical properties of juices used in the validation of PEF treatments.

Property	Carrot Juice	Apple Juice
pH	5.94 ± 0.01	3.40 ± 0.01
°Brix	6.35 ± 0.05	11.9 ± 0.1
Conductivity	4.3 ± 0.01	1.64 ± 0.01

**Table 2:** Coefficients and F-values of the mathematical equation to describe the influence of electric field strength and treatment time on the *Cryptosporidium* oocysts’ permeabilization to PI after multiple regression modelling. Statistics to test the adequacy are also shown.

	Sum of Squares	df	Mean Square	F-Value	*p* Value
Model	40,577.82	4	10,144.46	101.82	<0.0001
**A**-Electric Field Strength	30,333.77	1	30,333.77	304.45	<0.0001
**B**-Treatment time	1677.53	1	1677.53	16.84	0.0002
**AB**	428.60	1	428.60	4.30	0.0437
**A^2^**	3949.28	1	3949.28	39.64	<0.0001
Residual	4583.20	46	99.63		
Lack of Fit	553.70	7	79.10	0.7656	0.6193
Pure Error	4029.50	39	103.32		

**Table 3:** Fit statistics of the model developed to describe the influence of electric field strength and treatment time on the *Cryptosporidium* oocysts permeabilization to PI.

Fit Statistics	
R^2^	0.8985
Adjusted R^2^	0.8897
Predicted R^2^	0.8767
Adeq Precision	27.0390

## Data Availability

The original contributions presented in the study are included in the article, further inquiries can be directed to the corresponding authors.
